# Identification of suitable reference gene and biomarkers of serum miRNAs for osteoporosis

**DOI:** 10.1038/srep36347

**Published:** 2016-11-08

**Authors:** Jian Chen, Kai Li, Qianqian Pang, Chao Yang, Hongyu Zhang, Feng Wu, Hongqing Cao, Hongju Liu, Yumin Wan, Weibo Xia, Jinfu Wang, Zhongquan Dai, Yinghui Li

**Affiliations:** 1 Institute of Cell and Development Biology, College of Life Sciences, Zhejiang University, Hangzhou, 3100058, China; 2 State Key Laboratory of Space Medicine Fundamentals and Application, China Astronaut Research and Training Center, Beijing, 100094, China; 3 Department of Endocrinology, Peking Union Medical College Hospital, Peking Union Medical College, Beijing, 100032, China

## Abstract

Our objective was to identify suitable reference genes in serum miRNA for normalization and screen potential new biomarkers for osteoporosis diagnosis by a systematic study. Two types of osteoporosis models were used like as mechanical unloading and estrogen deficiency. Through a large-scale screening using microarray, qPCR validation and statistical algorithms, we first identified miR-25-3p as a suitable reference gene for both type of osteoporosis, which also showed stability during the differentiation processes of osteoblast and osteoclast. Then 15 serum miRNAs with differential expression in OVX rats were identified by microarray and qPCR validation. We further detected these 15 miRNAs in postmenopausal women and bedrest rhesus monkeys and evaluated their diagnostic value by ROC analysis. Among these miRNAs, miR-30b-5p was significantly down-regulated in postmenopausal women with osteopenia or osteoporosis; miR-103-3p, miR-142-3p, miR-328-3p were only significantly decreased in osteoporosis. They all showed positive correlations with BMD. Except miR328-3p, the other three miRNAs were also declined in the rhesus monkeys after long-duration bedrest. Their AUC values (all >0.75) proved the diagnostic potential. Our results provided a reliable normalization reference gene and verified a group of circulating miRNAs as non-invasive biomarkers in the detection of postmenopausal- and mechanical unloading- osteoporosis.

Osteoporosis is a systemic skeletal disorder associated with a reduction of bone mass and deterioration of microarchitecture, which increases bone fragility and the risk of fractures^1^. Bone homeostasis is a dynamic equilibrium associated with bone formation mediated by osteoblasts and bone resorption mediated by osteoclasts. The complex regulation processes are controlled by many factors, including hormones, cytokines and mechanical stimulation etc. Estrogen deficiency which mainly occurred in postmenopausal women and mechanical unloading owing to long-duration bedrest or exposure to microgravity are two main types of osteoporosis in clinical practice.

microRNAs (miRNAs) are a class of endogenous, single stranded non-coding RNAs with the length of approximately 22 nucleotides, which are widely expressed in higher organisms and regulate gene expression at post-transcriptional level. miRNAs play important roles in bone homeostasis, such as the differentiation regulation of osteoblast and osteoclast^2^,^3^. Recently, several groups reported miRNAs circulated in highly stable, cell-free form in body fluids including serum and plasma^4^,^5^,^6^,^7^. Because they met the three fundamental criteria of valuable biomarker: measurability, validation and utility, circulating miRNAs had great potential to serve as noninvasive biomarkers for molecular diagnostics^8^,^9^. Only recently, results from circulating miRNAs analysis in patients with osteopenia, osteoporosis and fragility fractures have been reported. Down-regulated miR-21 and up-regulated mir-133a were suggested as sensitive plasma biomarkers for postmenopausal osteoporosis. Both miRNAs showed significant moderate to strong correlations with BMD (bone mineral density)^10^. From 4 miRNAs which were slight differential expression between low and high BMD women, Cao *et al.* demonstrated miR-422a was significantly up-regulated in the low BMD group compared to the high BMD group^11^. Moreover, Seeliger *et al.* reported that five miRNAs (miR-21, miR-23a, miR-25, miR-100 and miR-125b) were increased in both serum and bone tissue in patients with acute osteoporotic fractures compared to non-osteoporotic fractures^12^. Weilner *et al.* showed that miR-328-3p and let-7g-5p were down-regulated in the serum of patients with osteoporotic fracture and could modulate the osteogenic differentiation of human mesenchymal stem cells *in vitro*^13^. Meanwhile, Panach *et al.* identified three up-regulated miRNAs (miR-122-5p, miR-125b-5p and miR-21-5p) were valuable biomarkers for osteoporotic fracture^14^. Due to the diverse nature of study designs, the number and type of regulated miRNAs identified varies significantly^9^. Specific and sensitive circulating miRNA biomarkers for postmenopausal osteoporosis has not been fully established and their potential of functioning as biomarkers for mechanical unloading induced osteoporosis remain unclear.

The gold standard approach for quantitative analysis of miRNAs is quantitative real time polymerase chain reaction (qPCR) due to its accuracy, sensitivity, specificity, reproducibility and robustness^15^. A reliable reference gene is highly important for the quantitative assay and is the basis of biomarker screening of circulating miRNAs. However, there is no current consensus on reference genes that are suitable for all serum miRNAs studies, which indicate that suitable reference genes should be verified in the individual experiment. Wang *et al.* identified snRNAU6, miR-92a and miR-16 and let-7a as internal reference gene group for qPCR normalization from osteogenesis imperfect patients^16^. Most of the results obtained by qPCR used different reference genes for normalization, such as miR-16, miR-93-5p in skeletal disease^10^,^12^,^13^,^14^. Although miR-93 had been defined as a plasma miRNA reference gene for tuberculosis^17^, it was also connected with osteoblast differentiation and bone mineralization^18^. External reference couldn’t correct for the other causes of variability such as the total concentration of miRNA fraction. Endogenous controls were required^19^. To our knowledge, no study has been involved in the evaluation of reference genes specific for serum miRNAs in osteoporosis. Therefore, a systematic study on the evaluation of both reference genes and biomarkers of osteoporosis was necessary.

In the present study, we employed a series of osteoporosis models to perform a systematic analysis of serum miRNA reference genes. miR-25-3p was identified as the most stable gene in both serum and bone tissue with or without osteoporosis and was recommended as an endogenous reference gene. Then, a group of circulating miRNAs were screened out and identified as potential biomarkers for osteoporosis from ovariectomy (OVX) rats, postmenopausal women and rhesus monkey with long-duration bedrest.

## Results

### Establishment and characterization of two osteoporotic models

**Figure 1:**
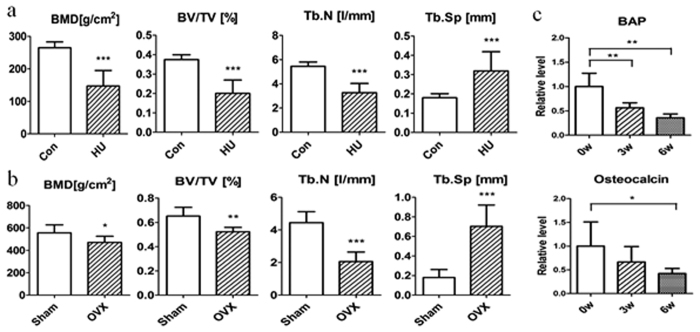
Characterization of bone phenotypes in two osteoporotic models. (**a**) The effects of hindlimb-unloading for 4 W on BMD and trabecular microarchitecture by determining with μCT (n = 6). (**b**) The effects of ovariectomy for 8 W on BMD and trabecular microarchitecture by determining with μCT (n = 6). (**c**) BAP and osteocalcin were detected in the serum of rhesus monkeys after bedrest for 0 w, 3w and 6 w (n = 6). All data are presented as mean ± SD. *p < 0.05, **p < 0.01, ***p < 0.001.

Mechanical unloading and estrogen deficiency were performed to induce osteoporosis by hindlimb-unloading (HU) male rats, head down bedrest monkeys and by OVX female rats respectively. Bone structural parameters were analyzed using μCT. After HU for 4 weeks, BMD, bone volume (BV/TV) and trabecular number (Tb.N) were substantially reduced, while trabecular separation (Tb.Sp) was significantly increased (Fig. 1a). After operation for 8 weeks, OVX rats showed similar results (Fig. 1b). The concentration of bone alkaline phosphatase (BAP) and osteocalcin of monkeys were significantly decreased after head down bedrest for 3 w and 6 w (Fig. 1c). These results indicated that the osteoporotic models were established successfully.

### Selection and identification of candidate reference genes

To screen reference genes of serum miRNAs in osteoporosis, rat miRNAs microarrays were performed using 3 pooled control samples and 4 pooled HU samples (each pool was composed of 4 independent serum sample with the same volume). All of the serum samples used in this study were passed through the hemolysis test. miRNAs were selected as candidate reference genes according to the following criteria: (1) highly expressed in all samples; (2) showed no differential expression between two groups (p > 0.05); and (3) less variation between inter- and intra-group, as measured by coefficient of variation. Therefore, ten miRNAs (miR-19b-3p, miR-21-5p, miR-25-3p, miR-30a-5p, miR-133b-3p, miR-140-5p, miR-150-5p, miR-199a-3p, miR-342-5p, miR-3473) were chosen as candidate reference genes. Meanwhile, two serum miRNAs (miR-16^20^,^21^ and let-7d^22^) reported highly stable in certain experimental condition were included to verify whether they were also stable in this study.

Then, the stability of the 12 candidate reference genes above mentioned were preliminary evaluated by qPCR. As shown in Table S1, the candidate reference genes displayed a wide range of expression level, with quantitative Cq ranging from 21 to 31. Three miRNAs (miR-21-5p, miR-30a-5p, miR-16-5p) showed significant differences between control group and HU group (p < 0.05). Therefore, these miRNAs were excluded from further stability analysis.

**Figure 2:**
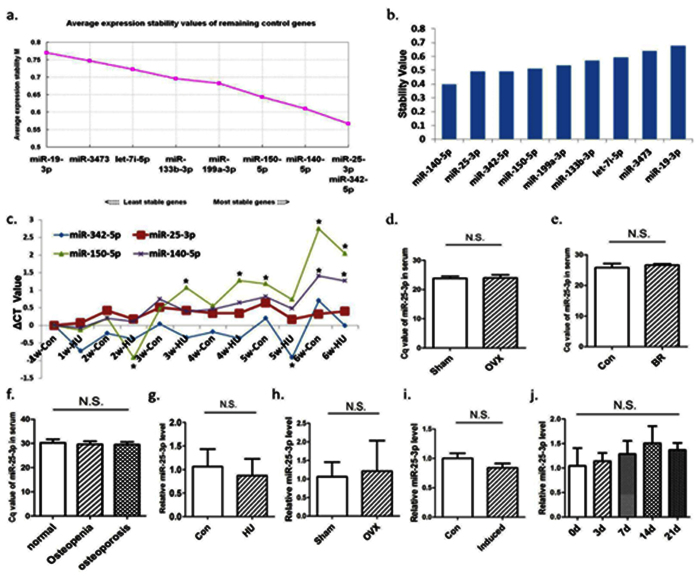
Identified miR-25-3p as the suitable reference gene for osteoporosis. (**a**) Ranking of candidate reference genes by geNorm, the most stable pair was recommended as miR-25-3p and miR-342-5p. (**b**) Ranking of candidate reference genes by NormFinder. (**c**) The expression pattern of candidate gene during hindlimb-unloading (HU) within 6 weeks, miR-25-3p showed as the most stable gene. *p < 0.05, HU. VS. CN, n = 6, N = 72. (**d**) miR-25-3p was detected in the serum of Sham and OVX rats (n = 12). (**e**) Rhesus monkeys with (n = 6) or without bedrest (n = 9). (**f**) Postmenopausal women with normal BMD (n = 19) or with osteopenic (n = 7) or with osteoporosis (n = 10). (**g**) miR-25-3p in the femur of hindlimb-unloading for 4 W (n = 6). (**h**) miR-25-3p in the femur of OVX for 8 W (n = 6). (**i**) miR-25-3p in BMMs after osteoclastic induction for 4d (n = 3). (**j**) miR-25-3p in primary osteoblasts after osteogenic induction for 0d, 3d, 7d, 14d and 21d (n = 4). All these showed no significant differences (N.S.) (p > 0.05). BR: Bedrest group, Con: control group.

Moreover, qPCR was carried out to analyze another serum sample set of HU and control rats (n = 18). The stability of the candidate reference genes (miR-19b-3p, miR-25-3p, miR-133b-3p, miR-140-5p, miR-150-5p, miR-199a-3p, miR-342-5p, miR-3473, let-7i-5p) was evaluated by two statistical algorithms: geNorm and Normfinder. geNorm calculates the gene expression stability value (M) by pairwise comparison of a particular gene with all other candidate genes, and the gene with the lowest M value is considered as the most stability^23^. By geNorm, miR-25-3p was ranked as the most stable gene and a combination of miR-25-3p, miR-342-5p and miR-140-5p was recommended for optimal normalization (Fig. 2a). NormFinder estimates not only the overall expression variation, but also both intra- and inter- group expression variation to evaluate the stability of candidate reference genes^24^. The most stable gene would be the lowest ranked. With this algorithm, miR-140-5p was identified as the most stable gene, followed by miR-25-3p (Fig. 2b). The ranking results of geNorm and NormFinder were summarized in Table S2. Obviously, miR-25-3p was rather stable in both algorithms, and was recommended as reference gene by comprehensive ranking.

### Validation of miR-25-3p as suitable reference gene in osteoporotic models

The stability of miR-25-3p was further validated at different stage of HU osteoporotic models in another independent experiment. Serum was collected from different stage at 1^st^ to 6^th^ week and the expression levels of 4 top ranked miRNAs (miR-25-3p, miR-140-5p, miR-342-5p and miR-150-5p) were assayed by qPCR. During HU within 6 weeks, miR-25-3p was the most stable gene and showed no significant difference between any pair matched groups (p > 0.05). Conversely, miR-140-5p, miR-342-5p and miR-150-5p were not as stable as miR-25-3p and showed some notable changes at some stage (Fig. 2c). Therefore, miR-25-3p was rather stable in serum during HU and could be considered as a suitable reference gene in this model.

To examine whether miR-25-3p is also stable in some extended osteoporotic models, including OVX rats, monkey after head down bedrest and postmenopausal women, serum was collected from each osteoporotic model. qPCR assay showed that there was no significant difference of miR-25-3p level between the sham and OVX rats, only with small variation of Cq value (Fig. 2d). The expression of miR-25-3p was also detected in the head down bedrest monkeys with osteoporosis due to mechanical unloading and showed no significant difference from that in the control groups (Fig. 2e). Similarly, no significant change of serum miR-25-3p expression was found among postmenopausal women with normal BMD, osteopenia or osteoporosis (Table 1 and Fig. 2f).

Furthermore, we postulated the serum reference gene for osteoporosis should also be stable in bone tissue and cells. Given this, miR-25-3p was detected in the femur of HU and OVX rats (Fig. 2gh). In addition, the expression pattern of miR-25-3p during osteoclast and osteoblast differentiation was investigated *in vitro*. BMMs were induced to osteoclastic differentiation by RANKL (receptor activator of nuclear factor κβ ligand) and M-CSF (Macrophage colony-stimulating factor) for 4 days and primary osteoblasts was induced to differentiation for 0, 3, 7, 14 and 21 days. The differentiation status was confirmed by TRAP staining for osteoclast or qPCR detection of osteoblastic gene for osteoblast (data not shown). The qPCR results of miR-25-3p expression showed no marked change compared with their control (Fig. 2i,j). All these results confirmed miR-25-3p was a suitable reference gene of serum miRNAs for osteoporosis.

### Expression difference of serum miRNA in OVX rats

**Figure 3:**
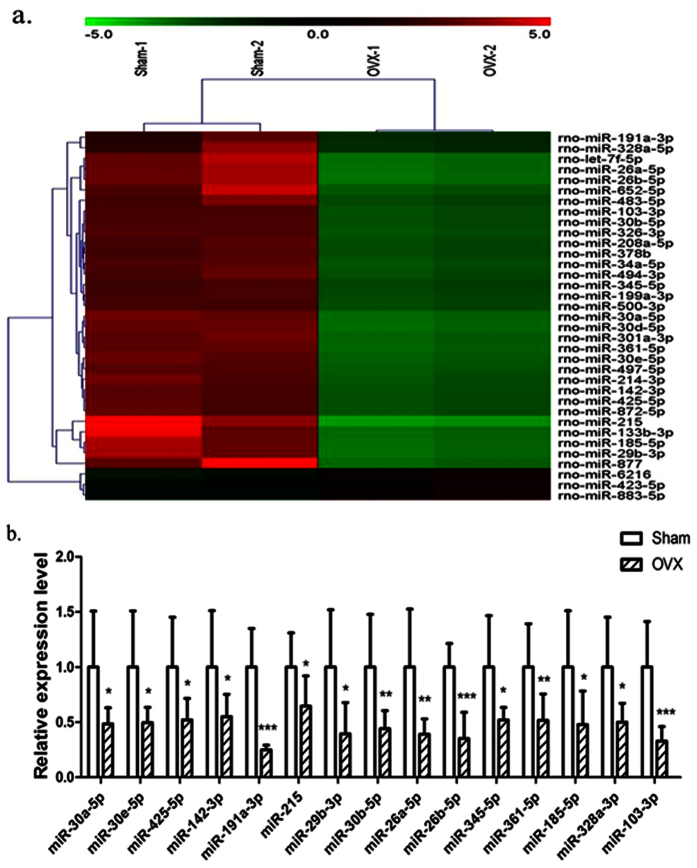
Analysis of differential expression of serum miRNAs in OVX rats. (**a**) miRNAs microarray was performed to analyze the expression of miRNAs in the serum of OVX rats, 3 up-regulated and 32 down-regulated-miRNAs were found in the serum of OVX rats compared with sham groups, the cutoff was a fold change > = 2 and p value < 0.05. (**b**) 15 down-regulated-miRNAs were confirmed by qPCR and were normalized to miR-25-3p (n = 8). *p < 0.05, **p < 0.01, ***p < 0.001, OVX VS. Sham.

To investigate the potential of serum miRNAs as biomarker for osteoporosis, serum miRNA microarrays were performed using 2 pooled sham samples and 2 pooled OVX samples (each pool was composed of 4 individual serum sample with the same volume). The threshold set for significantly changed genes was a fold change ≥2 and p value ≤ 0.05. As shown in Fig. 3a, 32 miRNAs were down-regulated and 3 miRNAs were up-regulated in the serum of OVX rats compared with the sham groups. Then, qPCR was performed to validate the differential expression of genes in an independent set of serum samples. The results showed that 14 miRNAs (miR-30a-5p, miR-30e-5p, miR-425-5p, miR-142-3p, miR-191a-3p, miR-215, miR-29b-3p, miR-30b-5p, miR-26a-5p, miR-345-5p, miR-361-5p, miR-185-5p, miR-103-3p) were down-regulated but no miRNA was up-regulated among above three altered miRNAs from microarray in OVX serum by normalizing to miR-25-3p (Fig. 3b). We also detected some miRNAs which was changed in women with osteoporosis or osteoporotic fracture. Only miR-328-3p was significantly down-regulated in OVX rat (Fig. 3b).

### Potential biomarkers for postmenopausal osteoporosis and mechanical unloading osteoporosis

**Figure 4:**
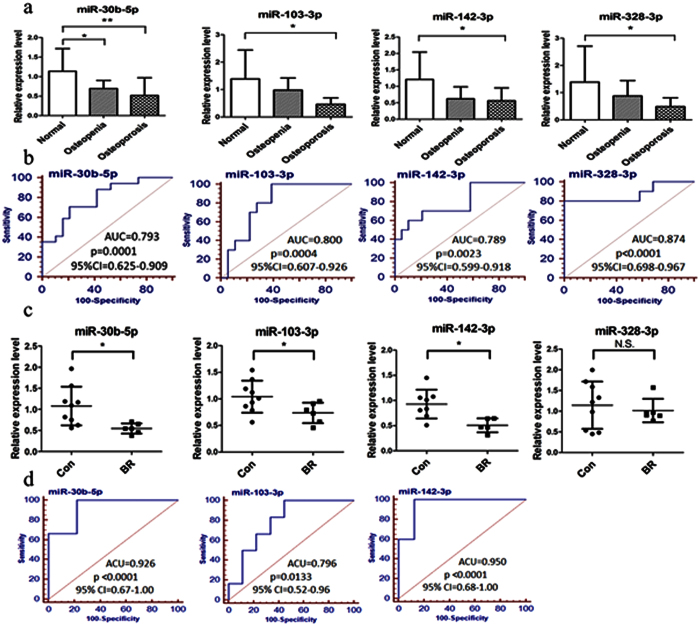
Potential biomarkers for postmenopausal osteoporosis and mechanical unloading induced osteoporosis. (**a**) miR-30b-5p was validated to be down-regulated in the serum of both osteopenic (n = 7) and osteoporotic (n = 10) patients compared with the normal BMD subjects (n = 19), miR-103-3p, miR-142-3p and miR-328-3p were validated to be down-regulation in the osteoporotic patients. *p < 0.05, **p < 0.01. (**b**) The potential diagnostic value of miR-30b-5p for bone loss (both osteopenia and osteoporosis), and miR-103-3p, miR-142-3p, miR-328-3p for osteoporosis was evaluated by ROC analysis. (**c**) Three miRNAs (miR-30b-5p, miR-103-3p, miR-142-3p) were validated to be down-regulated in the serum of rhesus monkeys with 42 days’ bedrest (n = 6) compared with the control group (n = 9). *p < 0.05. (**d**) The potential diagnostic value evaluated by a ROC analysis. AUC = area under the curve, CI = confidence interval.

In order to validate the potential of serum miRNAs as clinical biomarker for osteoporosis, the expression levels of above 15 miRNAs were measured by qPCR and ROC analysis was performed on the data from postmenopausal women with normal BMD (T-score > −1), osteopenia (−2.5 > T-score > −1) or osteoporosis (T-score < −2.5). The clinical characteristics of the subjects were shown in Table 1. Among the detected miRNAs, miR-30b-5p showed significant down-regulation in both osteopenic and osteoporotic patients, while miR-103-3p and miR-142-3p were markedly down-regulated only in osteoporotic patients (Fig. 4a). In addition, miR-328-3p also showed a notable decrease in osteoporotic patients in our study by normalizing to miR-25-3p (Fig. 4a), which was reported down-regulation in the serum of postmenopausal osteoporotic fracture and involved in bone formation by Weilner *et al.*^13^. To access the potential diagnostic value of miR-30b-5p for bone loss (osteopenia and osteoporosis), and the value of miR-103-3p, miR-142-3p, miR-328-3p for osteoporosis, ROC analysis was conducted and the associated area under the curve (AUC) was used to confirm the diagnostic value of each miRNA. As shown in Fig. 4b, the AUC of miR-30b-5p was 0.793 (95% CI = 0.625–0.909, p = 0.0001), and 0.800 for miR-103-3p (95% CI = 0.607–0.926, p = 0.0004), 0.789 for miR-142-3p (95% CI = 0.599–0.918, p = 0.0023), and 0.874 for miR-328-3p (95% CI = 0.698–0.967, p < 0.0001). The sensitivity and specificity evaluated with optimal cutoff points were shown in Table 2. Then, the multiple correlation analysis was performed between these four miRNAs and BMD. After adjusting for age, weight and height, miR-30b-5p, miR-103-3p, miR-142-3p and miR-328-3p were significantly positively correlated with H-BMD (total hip BMD) (r = 0.541, p = 0.001; r = 0.355, p = 0.039; r = 0.650, p < 0.001; r = 0.355, p = 0.039 respectively). Meanwhile, miR-30b-5p and miR-142-3p were significantly associated with FN-BMD (femur neck BMD) (r = 0.439, p = 0.009; r = 0.489, p = 0.003 respectively) as well.

To investigate whether these four miRNAs could also function as potential biomarkers for mechanical unloading osteoporosis, we detected them in the serum of rhesus monkeys with or without 42 days’ bedrest. As shown in Fig. 4c, miR-30b-5p, miR-103-3p and miR-142-3p were significantly down-regulated after bedrest, while miR-328-3p had no significant change. And the ROC analysis (Fig. 4d) showed considerable diagnostic value: 0.926 for miR-30b-5p (95% CI = 0.67–1.00, p < 0.0001), 0.796 for miR-103-3p (95% CI = 0.52–0.96, p = 0.0133), 0.950 for miR-142-3p (95% CI = 0.68–1.00, p < 0.0001). The sensitivity and specificity evaluated with optimal cutoff points were shown in Table 2.

## Discussion

The expression patterns of circulating miRNAs have been shown to correlate with the occurrence and progression of human diseases^8^,^25^. Recently, the diagnostic potential of circulating miRNAs for postmenopausal osteoporosis has been investigated and some miRNAs were identified as potential biomarkers^10^,^12^,^13^,^14^. Because of the diverse study designs and models, more studies were required to establish circulating miRNAs biomarkers for osteoporosis with high specificity and sensitivity. For example, no comprehensive investigation on reference genes for osteoporosis has been performed which is indispensable for quantitative analysis.

In the present study, we first filtered candidate reference genes in HU osteoporosis model using miRNAs microarray and qPCR validation. miR-25-3p was recommended as the most suitable reference gene according to the ranking of 9 candidate miRNAs by two statistical algorithms (geNorm and NormFinder). To extend its application scope, we further estimated the stability of miR-25-3p in various osteoporosis models and species. Osteoporosis is a systematic skeletal disease caused by complex causes, including estrogen deficiency which mainly occurred in postmenopausal women and mechanical unloading owing to long-duration bedrest or exposure to microgravity^26^,^27^. In the OVX and HU rat models, it was validated to be stable between intra- or inter- groups. miR-25-3p was also detected in rhesus monkeys after long-duration bedrest and postmenopausal women with osteoporosis. Results showed that there were no significant differences of miR-25-3p expression in those paired subjects. Afterwards, we postulated that the serum reference gene for osteoporosis should also be stable in bone tissue, the differentiation processes of osteoblast and osteoclast. Indeed, miR-25-3p showed no marked alterations in the femur of OVX or HU rats, as well as during osteogenic or osteoclastic differentiation. Recently, Das *et al.* reported miR-25-3p was the most suitable reference gene in human cancer cell lines and even more stable than RNU6^28^. Ssa-miR-25-3p was identified as the most suitable single reference gene in Atlantic salmon^29^. Moreover, our results conformed the finding of Seeliger *et al.*^12^ who reported no differential expression of miR-25-3p in serum between non-osteoporotic and osteoporotic fracture patients. Therefore, the consensus of the unchanging expression signature of miR-25-3p in serum proved that miR-25-3p should be a suitable reference gene of serum miRNAs for osteoporosis.

The potential of serum miRNAs as clinical biomarkers for postmenopausal osteoporosis and mechanical unloading induced osteoporosis was investigated. miRNAs microarray screened several up- or down-regulated miRNAs in the serum of OVX rats. 14 of these down-regulated miRNAs and miR-328-3p reported previously were verified by qPCR. And 3 of them (miR-30b-5p, miR-103-3p and 142-3p) were finally confirmed down-regulation in the serum of both OVX rats, postmenopausal osteoporotic patients and bedrest monkeys. These miRNAs showed the valuable biomarkers by a ROC analysis.

miR-30b-5p and miR-103-3p were two osteogenesis-related miRNAs. Eguchi *et al.* reported that miR-30b-5p expression decreased at the late stage of osteogenic induction and Runx2 was predicted as one of its target genes^30^. Balderman *et al.* showed that BMP2 increased Runx2 expression in vascular smooth muscle cells and promoted calcification through down-regulating miR-30b-5p^31^. miR-103-3p, identified as a mechanosensitive and bone abundant miRNA, inhibited the differentiation of osteoblasts by directly targeting Runx2^32^ and the proliferation of osteoblasts by targeting Cav1.2^33^. It is interesting that these osteogenesis-inhibitory miRNAs were down-regulated in the serum of osteoporotic patients and bedrest monkeys. We presumed the possible reasons as following: Firstly, miR-30b-5p and miR-103-3p were identified as ostemiRs in osteoblasts and osteocytes^30^, while postmenopausal osteoporosis was mainly due to the over-activity of osteoclasts^34^. Secondly, it had been shown that bone loss was partly compensated by increased osteobalstogenesis during estrogen deficiency^35^. miR-30b-5p and miR-103-3p may involve in these processes. Finally, it maybe resulted from the selective package and secretion process of circulating miRNA. Previous reports showed that the expression pattern of some miRNAs in intracellular was contrary to that in extracellular owing to the selective disposal^36^,^37^,^38^.

miR-142-3p was also an important ostemiR. Its expression was markedly increased during osteogenic differentiation in hFOB1.19 cells. It promoted osteoblast differentiation by targeting APC to activate Wnt signaling^39^. Moreover, miR-142-3p also acted as an essential modulator in macrophage differentiation^40^. As is well known, osteoclasts were formed by the fusion of circulating mononuclear precursor cells in the presence of RANKL and M-CSF. It was attractive to identify the important role of miR-142-3p in osteoclast differentiation.

Interesting, miR-328-3p showed significant down-regulation in the serum of OVX rats and postmenopausal osteoporotic patients, but no significant change in bedrest monkeys. Weilner *et al.* reported that miR-328-3p was down-regulated in the serum of postmenopausal osteoporotic fracture patients^13^, which was consistent with our results. The knockdown of miR-328-3p in adipose-derived mesenchymal stem cells significantly reduced ALP activity during osteogenic differentiation^13^. It had been shown that miR-328-3p potentially activated WNT signaling by repression of the WNT-inhibitor SFRP-1 (secreted Frizzled-related protein 1)^41^, which also played an important role in both osteoblast and osteoclast differentiation^42^,^43^. In addition, CD44 was another experimentally validated target gene of miR-328-3p^44^ and its deficiency significantly inhibited osteoclast activity^45^. However, the regulation mechanisms of miR-328-3p on bone metabolism remain unclear, we speculated miR-328-3p may involve in the different mechanisms between postmenopausal osteoporosis and mechanical unloading induced osteoporosis.

Circulating miRNA as a new type of biomarker has shown its powerful potential in different diseases, because of its simplicity of obtaining serum sample and facility to be measurement. In present, we verified miR-25-3p as a stable reference endogenous control gene and 4 downregulated biomarkers from women with normal BMD, osteopenia and osteoporosis. Three serum miRNA (miR-103-3p, miR-142-3p and miR-328-3p) were markedly down-regulated only in osteoporotic patients and miR-30b-5p decreased in both osteopenic and osteoporotic patients. The limitations of this study are the small size of the bone loss (osteopenia and osteoporosis) patients. If These results are able to be demonstrated by a larger population of bone loss in the future, the combination of these miRNA could detect the progress and status of osteoporosis and could be applicate in clinical practice.

In conclusion, we presented a systematic study on the screening of the reference gene and biomarker of serum miRNAs for osteoporosis. miR-25-3p was identified as the most suitable reference gene and expanded its application scope to both estrogen deficiency- and mechanical unloading- induced osteoporosis. Then, a group of circulating miRNAs were identified as a non-invasive biomarker in postmenopausal osteoporosis and mechanical unloading induced osteoporosis. These findings may provide a non-invasive methodology for the diagnosis of clinical osteoporosis.

## Materials and Methods

### Study Subjects

Randomly selected postmenopausal patients from Peking Union Medical College Hospital were recruited into this study. Menopause was defined as the absence of menstruation for at least one year. Exclusion criteria were according to the previous study^46^: smoking history, chronic liver and renal diseases, significant gastrointestinal diseases such as inflammatory bowel disease and chronic diarrhea, metabolic or inherited bone disease, and corticosteroid, anticonvulsant or anti-tuberculosis therapy for more than 6 months during the previous year. BMD of the femur neck (FN-BMD) and total hip (H-BMD) was measured by DXA scan. Written informed consent was obtained from all participants and all methods were carried out in accordance with the protocol approved by the Ethics Committee of Peking Union Medical College Hospital.

### Animal experiments

All animal protocols were performed in accordance with the standard ethical guidelines and approved by the Institutional Review Board of China Astronaut Research and Training Center. SD Rats were purchased from the Animal Center of the Academy of Military Medical Sciences (Beijing China). Male rats aged 7 weeks were allowed to acclimatize their new surrounding for 1 week as singletons and free access to water and standard chow under a 12/12 h light/dark cycle. Subsequently, the rats were randomly assigned to the control group (Con) and the HU group. The experimental manipulation followed the methodology of Wronski and Morey-Holton^47^. Briefly, the rat was suspended by the tails using a strip of adhesive surgical tape attached to a chain hanging from a beam. The rats were 30° angle to the floor with only the forelimbs touching the floor and allowed to move freely to food and water.

Female SD rats aged 12 weeks were acclimatized to new surrounding for 1 week, and then randomly assigned to the sham-operated group (sham) and the OVX group according to body weight. 8 weeks after operation, all rats were euthanized for bloods collection and femur bones dissection.

A 42-day bedrest experiment was performed with rhesus monkey. During the bed-rest, the healthy monkeys with body weight of 5 to 8 kg and 4–8 years old purchased from institute of Beijing Xieerxin Biology Resource, were remained −10-degree head-down tilt position for 6 weeks and were supervised and monitored 24 h per day.

### Blood collection

At the end of the experiments, monkey’s blood was collected from femoral vein at 3^rd^ week and 6^th^ week. The concentration of BAP and osteocalcin were detected by RIA (Immunodiagnostic Systems Limited (IDS), UK) and ELISA (R&D System, USA) respectively. Rats were euthanized with an overdose chloral hydrate, and blood was collected by cardiac puncture. The whole blood was separated into serum and cellular fractions by centrifugation at 2000 g for 10 min at 4 °C, followed by a 20 min high-speed centrifugation at 12000 g at 4 °C to completely remove cell debris. The entire process was accomplished within 2 h and the supernatant serum was stored at −80 °C until analysis.

### Quality control of serum samples by qPCR detection

For the discovery study sample, RNA quality and process procedure were checked during the analysis process. To avoid the effects of erythrocytolysis, a hemolysis index (difference between miR-23a-3p and miR-451a-5p) was computed based on the qPCR detection according to previous report^48^. Only the samples with ΔCT value (miR-23a-3p – miR-451a) <7 were used in the following analysis. For the quality control for the difference in RNA extraction or RT efficiencies, a synthetic cel-miR-39 was utilized as spike-in control RNA, which was useful for the assessment of technical variability, but not for biology normalization^49^. cel-miR-39 was spiked with the same quantity prior to miRNA isolation and analyzed by qPCR to ensure consistent RNA isolation. The stable threshold cycle (Cq) values of cel-miR-39 obtained from all spike-ins indicated successful RNA isolation, reverse transcription and qPCR detection system.

### μCT analysis

The femur bone was scanned *ex vivo* using an μCT system vivaCT40 (SCANCO medical AG, Switzerland) with an isotropic voxel size of 10.5 μM. The 3D reconstruction of the mineralized tissue was performed automatically by the system. About 200 slices of distal femora metaphysis was chosen for analysis of the microarchitecture parameters, including BMD, BV/TV, Tb.N and Tb.Sp.

### Cell culture

Primary osteoblasts were isolated from the calvaria of newborn rats. Briefly, the dissected calvaria bone was minced and subjected to sequential digestion with 0.25% trypsin (Gibco, USA) for 10 min and then with 0.2% collagenase II (Sigma, USA) for 60 min. The cells were collected and cultured in Alpha Modified Eagle’s Medium (α-MEM, Hyclone, USA) containing 10% FBS (Gibco, USA) with 1% penicillin/streptomycin under 5% CO_2_ at 37 °C. The culture medium was changed every other day and primary osteoblast in passage 3 or 4 were used for the following experiments. Osteogenic induction was performed by replacing the medium with osteogenic medium (containing 10 nM dexamethasone, 10 mM β-glycerophosphate, and 50 μg/mL vitamin C) (Sigma, USA). The osteogenic medium was changed every other day.

Osteoclasts were induced from bone marrow monocytes (BMMs) with M-CSF and RANKL (R&D Systems, USA). Bone marrow cells were isolated and cultured in α-MEM containing 10% FBS with 1% penicillin/streptomycin and M-CSF (10 ng/ml) for 1 day. Non-adherent cells were considered as BMMs, and cultured in α-MEM containing 10% FBS, M-CSF (30 ng/ml) and RANKL (50 ng/ml) under 5% CO2 at 37 °C. The culture medium was changed every other day.

### RNA isolation and quantitative RT-PCR

Bone tissue and cell miRNAs were isolated by miRNeasy Mini Kit (QIAGEN, Hilden, Germany) according to the manufacturer’s instructions. For serum miRNAs, the method was similar to the previously described^4^. In briefly, pipetted 200 μL serum sample accurately were added with 600 μL RNAiso for small RNA (Takara, Dalian, China). Cel-miR-39 was spike-in prior to RNA isolation to act as an additional quality control. Then miRNAs were isolated according to the manufacturer’s instructions, followed by reverse transcription with SYBR PrimeScript^TM^ miRNA RT-PCR Kit (Takara, Dalian, China). qPCR was conducted in a total volume of 20 μL using SYBR Premix Ex Taq^TM^ Kit (Takara, Dalian, China) and the PCR cycling program included 95 °C for 2 min, 40 cycles of 95 °C for 10 s, 60 °C for 20 s, 72 °C for 20 s, melting curve analysis was carried out at the end of cycling program. The qPCR was performed in triplicate on LightCycler^®^96 (Roche, Switzerland).

### miRNA microarray

miRNA microarray was completed by Oebiotech (Shanghai, China). Briefly, total RNA was quantified by the NanoDrop ND-2000 (Thermo Scientific) and the RNA integrity was assessed using Agilent Bioanalyzer 2100 (Agilent Technologies). The sample labeling, purification, microarray hybridization and washing were performed based on the manufacturer’s standard protocols, recommended agents and devices. After washing, the arrays (Agilent Rat miRNA 8*60 K, Design ID: 046066) were scanned with the Agilent Scanner G2505C. Feature Extraction software (version 10.7.1.1, Agilent Technologies) was used to analyze array images to get raw data. Next, Genespring software (version 12.5; Agilent Technologies) was employed to finish the basic analysis of the raw data. Differentially expressed miRNAs were then identified through fold change (≥2) as well as p value (≤0.05) calculated using t-test.

### Data analysis

Expression levels of the candidate reference gene were determined by Cq value. Serum miRNA were determined using the relative quantification method (2^−ΔΔCt^) with the selected reference gene. All generated quantitative data was presented as the mean ± SD and difference were analyzed with One-way ANOVA or two-way ANOVA. Multiple correlation analysis was performed between miRNAs expression levels and BMD with adjustment for age, weight and height. Statistical analyses were performed using SPSS 19.0, p < 0.05 was considered as statistical significance for all tests. Two statistical algorithms including geNorm and Normfinder were used to compare gene stability among candidate reference genes according to the author’s instructions^23^,^24^. Receiver operating characteristic (ROC) analysis was performed by Medcalc.

## Additional Information

**How to cite this article**: Chen, J. *et al.* Identification of suitable reference gene and biomarkers of serum miRNAs for osteoporosis. *Sci. Rep.* **6**, 36347; doi: 10.1038/srep36347 (2016).

**Publisher’s note:** Springer Nature remains neutral with regard to jurisdictional claims in published maps and institutional affiliations.

## Supplementary Material

Supplementary Information

## Figures and Tables

**Table 1:** Characteristics of the human subjects.

	Normal (n = 19)	Osteopenia (n = 7)	Osteoporosis (n = 10)	p value
Age	51.89 ± 2.56	72.86 ± 7.73	77.4 ± 4.45	<0.001
FN-BMD (g/cm2)	1.06 ± 0.07	0.79 ± 0.04	0.58 ± 0.08	<0.001
FN T-score	1.16 ± 0.63	−1.20 ± 0.28	−2.73 ± 0.68	<0.001
H-BMD (g/cm2)	1.13 ± 0.09	0.77 ± 0.05	0.61 ± 0.08	<0.001
H T-score	1.48 ± 0.86	−1.34 ± 0.37	−2.70 ± 0.69	<0.001
Ca (mmol/L)	2.40 ± 0.12	2.46 ± 0.11	2.46 ± 0.14	N.S.
P (mmol/L)	1.18 ± 0.14	1.24 ± 0.15	1.23 ± 0.15	N.S.
CTX (ng/ml)	0.39 ± 0.15	0.73 ± 0.22	0.54 ± 0.24	0.001

**Table 2:** Sensitivity and specificity evaluated with optimal cutoff points.

miRNA	For postmenopausal osteoporosis		For mechanical unloading induced osteoporosis	
	Sensitivity (%)	Specificity (%)	Sensitivity (%)	Specificity (%)
miR-30b-5p	70.59	78.95	100	77.78
miR-103-3p	80	72.22	66.67	77.78
miR-142-3p	70	78.95	100	87.5
miR-328-3p	80	100	—	—
