# Supplementary material for: Identification of suitable reference gene and biomarkers of serum miRNAs for osteoporosis
Source: Sci Rep. 2016 Nov 8;6:36347. doi: 10.1038/srep36347 (PMC5099566; doi:10.1038/srep36347)
Supplement: Supplementary Information [file srep36347-s1.doc]

**Identification of suitable reference gene and biomarkers of serum miRNAs for osteoporosis**

Jian Chen^1,2#^, Kai Li^2#^, Qianqian Pang^3^, Chao Yang^2^, Hongyu Zhang ^2^, Feng Wu^2^, Hongqing Cao ^2^, Hongju Liu^2^, Yumin Wan^2^, Weibo Xia^3*^, Jinfu Wang^1*^, Zhongquan Dai^2*^, Yinghui Li^2^

1. Institute of Cell and Development Biology, College of Life Sciences, Zhejiang University, Hangzhou, 3100058, China; 2. State Key Laboratory of Space Medicine Fundamentals and Application, China Astronaut Research and Training Center, Beijing, 100094, China; 3. Department of Endocrinology, Peking Union Medical College Hospital, Peking Union Medical College, Beijing, 100032, China.

Table S1. Expression level of candidate reference genes

| Name | Control (Cq ± SD) | HU (Cq ± SD) | p value |
| --- | --- | --- | --- |
| miR-19b-3p | 25.99±0.75 | 25.40±1.15 | 0.241 pass |
| miR-21-5p | 25.10±0.46 | 24.29±0.84 | 0.032 fail |
| miR-25-3p | 21.36±0.21 | 21.54±0.33 | 0.191 pass |
| miR-30a-5p | 23.51±0.52 | 22.74±0.76 | 0.034 fail |
| miR-133b-3p | 28.67±0.41 | 29.00±0.46 | 0.154 pass |
| miR-140-5p | 26.67±0.42 | 26.61±0.77 | 0.849 pass |
| miR-150-5p | 22.61±0.77 | 22.34±0.25 | 0.063 pass |
| miR-199a-3p | 30.26±0.68 | 30.43±0.32 | 0.514 pass |
| miR-342-5p | 23.66±0.63 | 23.19±0.60 | 0.150 pass |
| miR-3473 | 22.72±0.38 | 22.18±0.68 | 0.071 pass |
| miR-16-5p | 22.80±0.63 | 21.85±0.55 | 0.006 fail |
| let-7i-5p | 27.87±1.03 | 27.55±0.68 | 0.481 pass |

Table S2. Comprehensive results by geNorm and Normfinder

|  | geNorm | |  | Normfinder | |
| --- | --- | --- | --- | --- | --- |
| Genes | M-value | Ranking |  | Stability value | Ranking |
| miR-25-3p | 0.567 | 1 |  | 0.491 | 2 |
| miR-342-5p | 0.567 | 2 |  | 0.492 | 3 |
| miR-140-5p | 0.610 | 3 |  | 0.399 | 1 |
| miR-150-5p | 0.643 | 4 |  | 0.512 | 4 |
| miR-199a-3p | 0.682 | 5 |  | 0.535 | 5 |
| miR-133b-3p | 0.696 | 6 |  | 0.569 | 6 |
| let-7i-5p | 0.722 | 7 |  | 0.592 | 7 |
| miR-3473 | 0.747 | 8 |  | 0.638 | 8 |
| miR-19-3p | 0.770 | 9 |  | 0.678 | 9 |
